# Atomistic Surface Passivation of CH_3_NH_3_PbI_3_ Perovskite Single Crystals for Highly Sensitive Coplanar-Structure X-Ray Detectors

**DOI:** 10.34133/2020/5958243

**Published:** 2020-09-22

**Authors:** Yilong Song, Liqi Li, Weihui Bi, Mingwei Hao, Yifei Kang, Anran Wang, Zisheng Wang, Hanming Li, Xiaohui Li, Yanjun Fang, Deren Yang, Qingfeng Dong

**Affiliations:** ^1^State Key Laboratory of Supramolecular Structure and Materials, College of Chemistry, Jilin University, Changchun 130012, China; ^2^State Key Laboratory of Silicon Materials and School of Materials Science and Engineering, Zhejiang University, Hangzhou 310027, China

## Abstract

Organic-inorganic halide perovskites (OIHPs) are recognized as the promising next-generation X-ray detection materials. However, the device performance is largely limited by the ion migration issue of OIHPs. Here, we reported a simple atomistic surface passivation strategy with methylammonium iodide (MAI) to remarkably increase the ion migration activation energy of CH_3_NH_3_PbI_3_ single crystals. The amount of MAI deposited on the crystal surface is finely regulated by a self-assemble process to effectively suppress the metallic lead defects, while not introducing extra mobile ions, which results in significantly improved dark current stability of the coplanar-structure devices under a large electric field of 100 V mm^−1^. The X-ray detectors hence exhibit a record-high sensitivity above 700,000 *μ*C Gy_air_^‐1^ cm^‐2^ under continuum X-ray irradiation with energy up to 50 keV, which enables an ultralow X-ray detection limit down to 1.5 nGy_air_ s^−1^. Our findings will allow for the dramatically reduced X-ray exposure of human bodies in medical imaging applications.

## 1. Introduction

High-performance X-ray detectors are increasingly important in many fields, including medical imaging, security monitoring, material inspection, and scientific research. [1–4] Especially in the medical diagnosis field, the X-ray-based medical imaging techniques such as X-ray radiography and computed tomography (CT) are gradually becoming the routine methods for disease diagnosis. However, in view of the cancer risk of high-dose X-ray radiation to the human body, [5, 6] X-ray detectors with much higher sensitivity are urgently needed to enable ultralow-dose X-ray imaging. For a semiconductor-type X-ray detector, which directly converts incident X-ray into electron-hole pairs, the sensitivity of the device is closely related to the attenuation capability and the electrical transport properties of the active material. This requires the material to possess both a high atomic number for large X-ray stopping power and a large mobility-lifetime (*μτ*) product to allow the efficient charge collection. [7] Amorphous Se- (*α*-Se-) based X-ray imaging sensor has occupied the majority market share of direct conversion-type X-ray detectors for decades. [8] However, its sensitivity is too low to enable ultralow-dose imaging, which is mainly limited by the small atomic number as well as the small *μτ* product of *α*-Se.

During the past few years, organic-inorganic halide perovskites (OIHPs) were identified as the promising new-generation candidate semiconductors for X-ray detection due to their outstanding physical and optoelectronic properties compared to the traditional X-ray detection materials like Si, CdZnTe, and *α*-Se, such as a large atomic number, [9–11] low intrinsic trap density, [12–14] and large *μτ* product. [9, 15, 16] Moreover, the low material cost and the low-temperature solution processability for high-quality perovskite single crystal (SC) greatly reduce the cost and complexity in device preparation and integration.

Currently, the best OIHP-based X-ray detector, which is reported by Huang et al., demonstrates a high sensitivity of 84,000 *μ*C Gy_air_^‐1^ cm^−2^ together with a low X-ray detection limit of 7.6 nGy_air_ s^−1^ for 8 keV X-ray, [16] which already significantly outperforms the commercial *α*-Se-based ones [8, 16]. However, one major hurdle that prevents the practical application of OIHP-based X-ray detectors is their ion migration issue, which is especially severe in iodine-based OIHPs like CH_3_NH_3_PbI_3_ (MAPbI_3_) due to the weaker chemical bond strength of Pb-I compared to Pb-Br and Pb-Cl [17–19]. The ion migration not only causes the dark current drift which increases the difficulty in signal reading and processing [20] but also leads to the formation of charge traps and even the decomposition of material [21, 22]. Although reducing the applied electric field can improve the bias stability of the device, it will sacrifice the collection efficiency of the charge carriers and hence the sensitivity of the device. Furthermore, the isotropic diffusion of the X-ray-generated carriers cannot be effectively inhibited with the small electric field, which eventually gives rise to the signal crosstalk between the adjacent pixels and hence impairs the X-ray imaging resolution. It is generally accepted that the major ion migration channel in OIHP SCs is through their surface, which contains a large amount of surface defects [17, 23]. Although the surface post treatment strategy has been widely used in perovskite solar cells to passivate the surface trap states, [24–28] it remains challenging to realize the complete suppression of ion migration in perovskite-based X-ray detectors. This is because in contrast to solar cells, the operation of X-ray detectors usually requires the application of large external bias in order to fully extract the X-ray-generated charge carriers from the thick active layer. In addition, the X-ray-generated carrier density in common X-ray detectors is usually several orders of magnitude lower than that in the solar cells under one sun illumination, which requires an ultralow surface trap density to avoid the photocurrent loss through surface recombination. Previously, the UV-ozone post treatment and the heteroepitaxial growth of BiOBr have been successfully used to passivate the defects in MAPbBr_3_ SCs and Cs_2_AgBiBr_6_ wafers, respectively, to realize the high-performance X-ray detectors. [9, 29] However, it is still urgently desired to explore an effective surface passivation method that is applicable to MAPbI_3_ SCs to suppress the ion migration under a large electric field.

Regarding the device structure, the present OIHP SC-based X-ray detectors mainly adopt the vertical sandwich-like structure [9, 11, 16, 30–33], while the devices with a coplanar-structure have seldom been reported. The major advantage of the coplanar-structure is that it differentiates the photosensitization length with the carrier transport distance. This is particularly advantageous for OIHP SC-based X-ray imaging sensors, which requires the preparation of large-area OIHP SC with preciously controlled thickness when adopting the vertical sandwich-like structure. In contrast, the charge collection efficiency of the coplanar-structure detector is insensitive to the active layer thickness (as long as it is larger than the attenuation length of the X-ray), which will greatly lower the requirement on the thickness uniformity of the perovskite SC and hence simplify the device fabrication procedure.

In this article, we show that the metallic lead defects on MAPbI_3_ SCs can be effectively eliminated with atomistic surface passivation by a controllable surface self-assemble process of methylammonium iodide (MAI) on the SC surface without excess MAI layer formation, which dramatically increases the bias stability of a coplanar-structure X-ray detector due to the suppressed ion migration. The detectors with atomistic surface passivation exhibit a record-large sensitivity above 700,000 *μ*C Gy_air_^‐1^ cm^−2^ under a large electric field up to 100 V mm^−1^ and are thus able to detect an ultralow X-ray dose rate down to 1.5 nGy_air_ s^−1^. The demonstrated devices, combining high sensitivity, low detection limit, and the superior robustness under large external electric field, show huge potential to high-resolution and low-dose X-ray medical imaging in the future.

## 2. Results and Discussion

### 2.1. Atomistic Surface Passivation in CH_3_NH_3_PbI_3_ SC X-Ray Detectors

**Figure 1:**
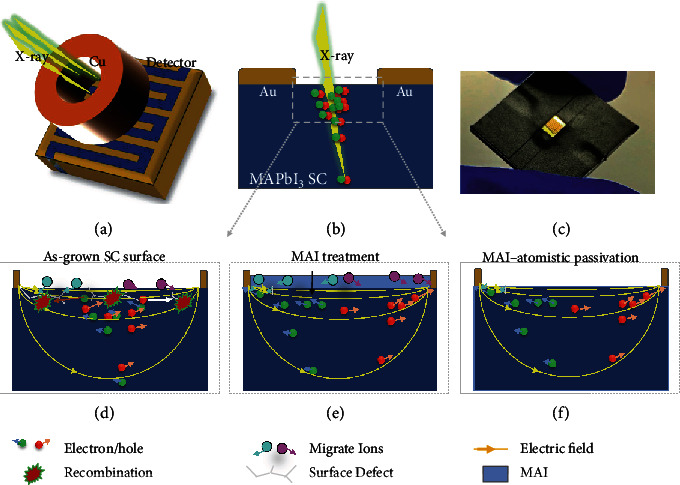
Schematic diagram of the device structure and working principle. (a, b) Top-view (a) and cross-sectional (b) schematic diagram of the coplanar-structure X-ray detector. (c) The photograph of a MAPbI_3_ single crystal coplanar-structure X-ray detector. (d–f) Schematic diagram of the working principle of the X-ray detector with different surface treatments: (d) untreated, (e) MAI treatment, and (f) MAI-atomistic passivation.

The coplanar-structure CH_3_NH_3_PbI_3_ SC X-ray detector was fabricated with a metal-semiconductor-metal architecture as shown in Figures 1(a)–1(c). The width of each finger of electrodes is approximately 50 *μ*m, and the spacing between the anode and cathode is approximately 50 *μ*m in interdigital electrodes, which was prepared simply by direct metal deposition through shadow masks on the SC surface, and does not require an expensive and complicated photolithography process. The device operation mechanism is schematically shown in Figure 1(b). The incident X-ray excites electrons and holes in the SC, which are separated by the applied external electric field and collected by the anode and cathode, respectively. Therefore, the charge collection efficiency is largely determined by the applied electric field. However, for regular OIHP-based detectors, usually, a small electric field is applied across the perovskite layer to avoid the ion migration effect for better operational stability. Although the use of SCs can eliminate the fast ion migration pathways caused by grain boundaries, the surface of the SC with a high density of defects can still behave as an ion migration channel, especially for the coplanar-structure device in which the electric field is located close to the crystal surface (Figure 1(d)). In our previous work, a significant passivation effect on the OIHP SC surface was proved by MAI treatment, which effectively recovered the surface damage of SCs with significantly suppressed surface trap density and longer carrier recombination lifetime and eventually led to efficient solar cell devices. [34] However, an excess of MAI, as shown in Figure 1(e), may introduce extra mobile ions on the SC surface, which is unfavorable in X-ray detectors which generally work under a much higher electric field than that of solar cells.

Here, the atomistic surface passivation, or an accurately controlled passivation of the SC surface without excess MAI layer formation (Figure 1(f)), was realized by a surface self-assemble process, in order to achieve surface passivation and suppress ion migration simultaneously. The atomistic passivation was realized simply by using a diluted MAI solution to cover the surface of the as-grown crystals followed by selective dissolving of the unbonded dissociative MAI with an isopropanol washing process, which was an excellent solvent to MAI but harmless to perovskites. Based on the atomic force microscope (AFM) images shown in Figure S1, the root mean square (RMS) roughness values of the untreated, MAI-treated, and atomistic passivated SCs were 2.14 nm, 1.46 nm, and 1.42 nm, respectively, which indicated that different processing conditions on the SC surface did not affect their surface roughness obviously.

**Figure 2:**
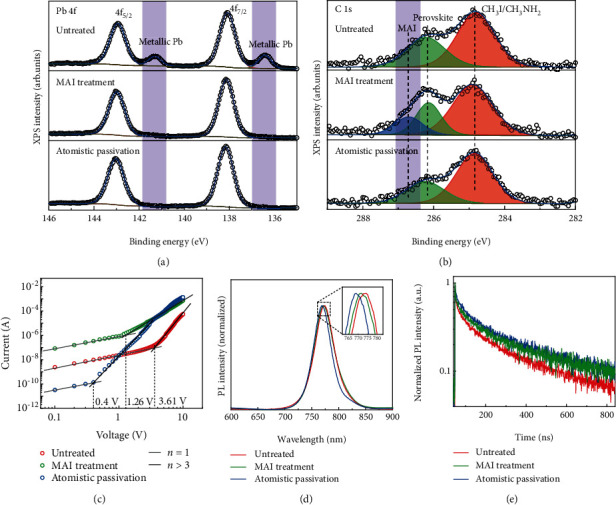
Atomistic surface passivation in MAPbI_3_ SCs. (a, b) XPS spectra corresponding to (a) Pb-4f and (b) C-1s core levels of the MAPbI_3_ SC surface with deferent treatments. (c) The SCLC measurement of the electron-only devices with different treatments. (d, e) The PL (d) and TRPL (e) spectra of the MAPbI_3_ SC surface with different treatments.

The atomistic surface passivation has proved to effectively passivate the surface defects and eliminate the excess MAI-rich layer based on the XPS measurement, which is sensitive to the surface properties of the specimen. As shown in Figures 2(a) and 2(b), there was plenty of metallic lead observed at the surface of as-grown MAPbI_3_ SCs, which can be passivated by MAI treatment that was consistent with our previous results. [34]. However, after regular MAI treatment, the peak related to metallic lead was suppressed, while the peak related to MAI emerged in the C1s spectrum, which indicated that an excess MAI layer was formed [35]. In contrast, by using the atomistic passivation instead of regular MAI treatment, both the peaks related to MAI and metallic lead were effectively suppressed. This was because MAI can interact with unbonded metallic lead during self-assemble treatment, and the subsequent isopropanol washing procedure can take away the excess unbonded MAI without destroying the bonded MAI.

The atomistic surface passivation effect was further evidenced by the significantly reduced surface trap density of SCs with passivation treatment based on the space-charge-limited current (SCLC) measurement as shown in Figure 2(c). The electron-only devices with the symmetric lateral structure of Cu/BCP/C60/MAPbI_3_ SC/C60/BCP/Cu were used for the SCLC measurement with the channel width of 50 *μ*m. From the *I* − *V* curve, we can obtain the trap filling threshold voltage for a gap-type structure by Geurst's SCLC model [36]:

(1) $$\begin{array}{c} V_{T}=\frac{\pi n_{\text{t}}L}{4\varepsilon_{0}\varepsilon_{\text{r}}}, \end{array}$$

where *n*_t_ is the surface trap density per unit area, *ε*_0_ is the vacuum permittivity, *ε*_r_ is the relative dielectric constant of MAPbI_3_ which equals to 32, [12] and *L* is the gap width. The surface trap density of SC with MAI treatment was suppressed from 1.63 × 10^10^ cm^‐2^ to 5.68 × 10^9^ cm^‐2^ and was further reduced to 1.8 × 10^9^ cm^‐2^ after isopropanol washing.

The passivation effect can also be observed from the photoluminescence (PL) spectrum and time-resolved photoluminescence (TRPL) measurements as shown in Figures 2(d) and 2(e), respectively. The PL peak located at 774 nm for untreated SCs, and there was a 3 nm blueshift of the PL peak for SCs with MAI treatment ,which was ascribed to the reduction of the band tail states of the SC surface. When the excess MAI was washed away, the PL peak blueshifts by 5 nm compared to the untreated ones due to the further trap states suppression. [26, 37] In accordance with the PL peak shift, the full width at half maxima (FWHM) of PL spectrum of SCs with atomistic passivation was reduced to 39.5 nm in comparison to that of 43.3 nm and 44 nm of the untreated and MAI-treated ones, respectively. In addition, from the single-log plots of the PL spectra (Figure S2), it was shown that the atomistic passivated SCs exhibited weaker band tail emission compared to the untreated and MAI-treated ones, indicating the significantly suppressed band tail states in the atomistic passivated SCs. The SCs with atomistic passivation also showed longer charge recombination lifetime (672.8 ns) than that of the untreated SCs (595.2 ns) and MAI-treated ones (655.2 ns), further confirming the trap passivation effect of the atomistic MAI treatment.

**Figure 3:**
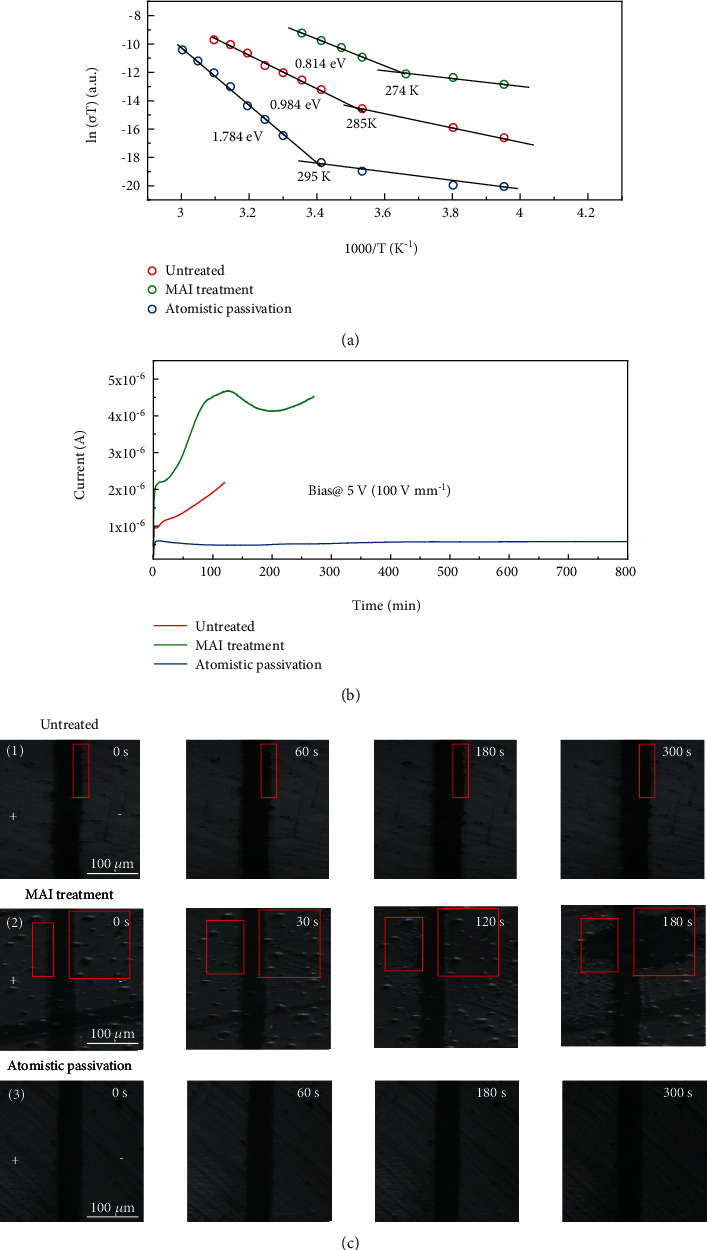
The characterization of the ion migration effect in MAPbI_3_ SCs. (a) Temperature-dependent conductivity of the MAPbI_3_ SC coplanar-structure devices in the dark. (b) The dark current as a function of time of the MAPbI_3_ SC coplanar-structure devices with different treatments at a constant bias of 5 V. (c) Microscope images of MAPbI_3_ single crystals with three treatment conditions under constantly applied bias (2 V *μ*m^−1^) at 25 mW cm^−2^.

The effective passivation without introducing excess MAI significantly reduces the ion migration effect on the surface of SCs, which is directly evidenced by measuring the variation of activation energy (*E*_a_) for the ion migration in MAPbI_3_ SCs with different treatments. The ion migration activation energy is obtained from the conductivity change with temperature of the SCs, which is a well-established method to evaluate the ion migration behavior in halide perovskites. [38–40] In the lower temperature range, the ionization energy is ascribed to the free charges, because most ions are frozen to move at a low temperature. While the activation energy derived at the higher temperature range is mainly contributed by the activation of ion migration. *E*_a_ is determined by measuring the *σ* as a function of temperature under a 0.4 V *μ*m^‐1^ electrical field (Figure 3(a)). The activation energy can be extracted from the Nernst-Einstein relation [38]:

(2) $$\begin{array}{c} \sigma \left(T\right)=\left(\frac{\sigma_{0}}{T}\right)\exp\left(-\frac{E_{\text{a}}}{k_{\text{B}}T}\right), \end{array}$$

where *k*_B_ is the Boltzmann constant, *σ*_0_ is a constant, and *T* is temperature. For the SCs without surface treatment, the ion conductivity began to dominate the total conductivity above 285 K in the dark, with an *E*_a_ of 0.984 eV. In comparison, the conductivity of SCs with MAI treatment showed the transition point at 274 K with an *E*_a_ of 0.814 eV. When the excessive MAI was washed away, the transition temperature was significantly increased to 295 K with a much higher *E*_a_ of 1.784 eV, which means an effectively suppressed ion migration at the surface of SCs by atomistic surface passivation.

The suppressed ion migration in atomistic surface passivated SCs was further supported by observing the morphology change of the crystal surface under a large electric field of 2 V *μ*m^−1^ with white light illumination of 25 mW cm^−2^, since the severe ion migration will cause the surface damage of perovskites [41, 42]. It was shown in Figure 3(c) that there was weak damage near the electrode for the untreated one, while the surface damage was much severe when there was excess MAI on the SC surface. As a strong contrast, no observable damage can be found on the atomistic surface passivated SCs under the same biasing condition, indicating the remarkably suppressed ion migration which was in accordance with the above *E*_a_ measurement results.

To evaluate the bias stability of SCs with atomistic passivation, the dark current of the devices was tracked at 5 V bias (100 V mm^−1^) with different surface treatment conditions (Figure 3(b)). Also, the excess MAI induced a significantly increased ionic current, which increased quickly and then decreased, indicating the accelerated ion migration effect compared to the untreated SCs. In contrast, the dark current of the devices was significantly reduced after the atomistic passivation due to the elimination of the extra MAI. Moreover, the SC devices with atomistic surface passivation showed dramatically enhanced dark current stability, which displayed no degradation even after 800 minutes under continuous biasing.

### 2.2. X-Ray Detector Characterization

**Figure 4:**
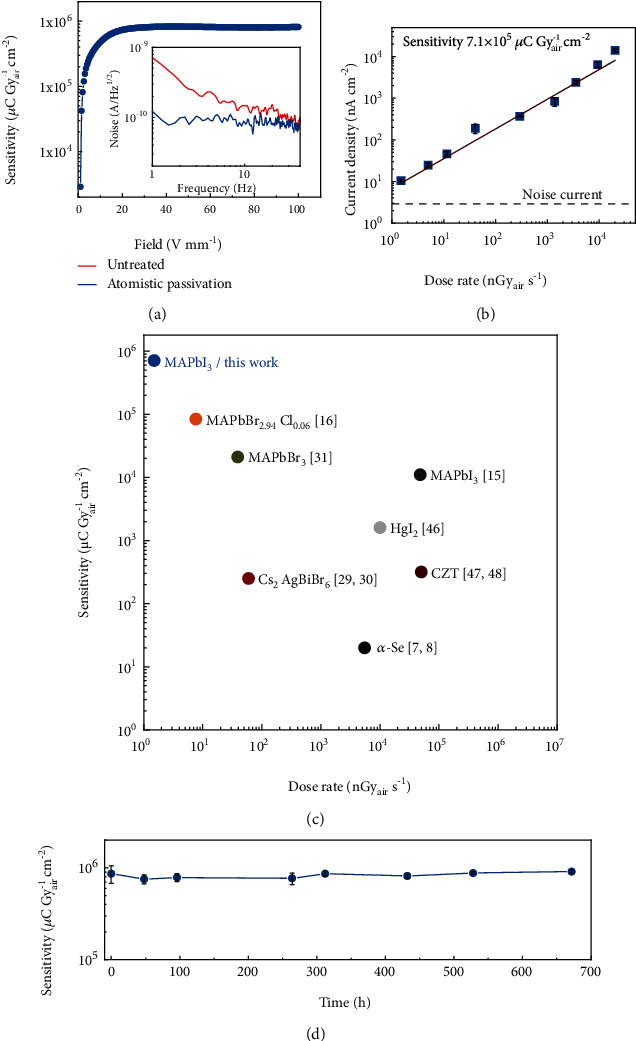
X-ray detection performance of the MAPbI_3_ SC devices with atomistic surface passivation. (a) Sensitivity versus electric field of the devices. Inset: the noise spectra of the devices without and the atomistic surface treatment. (b) The current density as a function of incident dose rate of the devices. The dashed line is noise current of the devices. (c) Summary of the sensitivity and detection limit of various kinds of X-ray detectors with different active materials. [7, 8, 15, 16, 29–31, 46–48] (d) The sensitivity changes of the devices with the storage time.

In view of the successful suppression of ion migration in MAPbI_3_ SC-based coplanar-structure devices with atomistic surface passivation, and the large attenuation coefficient of the high-energy X-ray in MAPbI_3_ SC (Figure S3), we exposed it to an X-ray source with an energy up to 50 keV and peak intensity at 22 keV to test its X-ray detection performance, which was collimated by a brass cylinder with a 2 mm diameter central hole in it. As shown in Figure 4(a), thanks to the excellent bias stability of the devices, a record-large sensitivity about 8 × 10^5^ *μ*C Gy_air_^‐1^ cm^‐2^ was achieved with a bias of 5 V applied across the 50 *μ*m wide channel under the X-ray dose rate of 20.3 *μ*Gy_air_ s^−1^. Since the device measurements were carried out in the air, it is crucial to evaluate the contribution from air ionization to the photocurrent of the devices. Therefore, we deposited the interdigitated Au electrodes with the same geometry on insulating glass substrates and measured the current response with successively tuning on and off the X-ray source. It was discovered that the photocurrent induced by air ionization was more than 4 orders of magnitudes smaller than that from the SC-based device (Figure S4), verifying the fidelity of the sensitivity of the device.

For X-ray detection application, noise is a very important figure of merit in addition to sensitivity, which was evaluated with a fast Fourier transform spectrum analyzer. We compared the noise spectra of the devices without and with atomistic MAI treatment and discovered that the MAI-atomistic-treated sample exhibited a decreased noise current of around 10^−10^ A Hz^-1/2^ which was is dominated by the 1/*f* noise in the low-frequency region (inset of Figure 4(a)). Since the 1/*f* noise is generally believed to originate from the charge trapping and detrapping processes in the conduction channel, the suppressed 1/*f* noise in the devices further confirms the remarkable surface passivation effect of MAI-atomistic surface treatment. [43–45]

The lowest detectable X-ray dose rate of the detectors is also an essential metric to evaluate the detection performance of the devices, especially for medical imaging application. To evaluate this, the X-ray dose rate was changed by adjusting the current of the X-ray tube or by adding Al foils as a filter. The photocurrent at different X-ray dose rates of the device at 5 V bias was shown in Figure 4(b). The average sensitivity of the device can be derived from the slope of the photocurrent density versus the X-ray dose rate, which was about 7.1 × 10^5^ *μ*C Gy_air_^‐1^ cm^‐2^. Notably, the current signal generated under the X-ray dose rate down to 1.5 nGy_air_ s^−1^ can be clearly differentiated from the noise current with the signal-to-noise ratio larger than 3, indicating its superior weak X-ray detection capability.

Figure 4(c) summarizes the sensitivity and the detection limit of various kinds of semiconductor-type X-ray detectors based on OIHPs and other materials. [7, 8, 15, 16, 29–31, 46–48] It is noted that the sensitivity of our devices is nearly 10 times larger than that of the best OIHP SC -X-ray detectors reported previously [16] and is also more than 35000-fold larger than that of the commercial *α*-Se X-ray detectors [8]. As a result of the significantly improved sensitivity, the detection limit of our devices is more than 5 times and 3600 times better than that of the OIHP SC and *α*-Se-based X-ray detectors, respectively [5, 8, 16], which displays great potential in ultralow-dose X-ray imaging application. The devices also exhibit good shelf stability with the sensitivity maintaining almost the same after storage in N_2_-filled glovebox for over 650 h (Figure 4(d)), indicating that the long-term stability of the device could be guaranteed with sophisticated encapsulation.

The superior X-ray detection performance largely originates from the suppressed surface ion migration effect of the MAI atomistic passivated MAPbI_3_ SCs, which enables us to apply a large electric field up to 100 V mm^−1^ on the device for charge extraction that is more than 20- to 50-fold stronger than that of the previously reported OIHP SC-based devices [9, 31, 49]. This is particularly important for MAPbI_3_ since it shows a much severer ion migration effect compared to MAPbBr_3_ and MAPbCl_3_. In addition, the electron-hole pair creation energy *W* of MAPbI_3_, which can be calculated according to the empirical model *W* = 1.43 + 2*E*_g_ (where *E*_g_ is the bandgap of the material) [50], is 77% of that of MAPbBr_3_. Thus, the photogenerated carrier density can be increased by 30% in MAPbI_3_ compared to MAPbBr_3_ under the same X-ray irradiation condition. Furthermore, the passivation of surface traps on MAPbI_3_SCs by atomistic passivation can elevate the charge extraction capacity under low-dose irradiation, which may otherwise be swallowed by the surface traps and thus deteriorate the detection limit of the device. Finally, the coplanar-structure device, which is firstly adopted in OIHP SC-based X-ray detectors, possesses the merit of differentiating the material thickness with the charge transport distance. This will facilitate the imaging array fabrication on the large-area MAPbI_3_ SCs irrespective of their thickness uniformity. Also, the carrier confinement capability of the coplanar-structure within the conductive channel can significantly inhibit the carrier diffusion and hence improve the X-ray imaging resolution in the future.

## 3. Conclusion

In summary, we have demonstrated a simple method to effectively passivate the metallic lead defects on the surface of MAPbI_3_ SCs, which is achieved by coating a thin atomic layer of MAI on the SC surface. The passivation of the crystal not only suppresses the surface charge recombination but also significantly increases the ion migration activation energy, which allows a large electric field of 100 V mm^−1^ to be applied on the coplanar-structure device with improved dark current stability. As a result, the optimized coplanar-structure X-ray detectors possess both a high sensitivity above 700,000 *μ*C Gy_air_^‐1^ cm^−2^ and an ultralow X-ray detection limit down to 1.5 nGy_air_ s^−1^, which remarkably outperform those of the previously reported perovskite as well as commercial *α*-Se-based X-ray detectors. The superior device performance makes it promising for the ultralow-dose X-ray medical diagnostic applications in the future. In addition, the results presented here provide an encouraging strategy to improve the bias stability of the perovskite SCs, which could also be applied in other perovskite-based optoelectronic devices like solar cells and light emitting diodes.

## 4. Materials and Methods

### 4.1. Materials

The materials used are as follows: lead iodide (PbI_2_) (99%, Born), methylammonium iodide (MAI) (99%, Born), gamma-butyrolactone (GBL) (99%, Aladdin), Fullerene-C60 (C60) (Xi'an Polymer Light), 2,9-dimethyl-4,7-diphenyl-1,10-phenanthroline (BCP) (Xi'an Polymer Light), and isopropanol (IPA) (≥99.5%, Sigma-Aldrich).

### 4.2. Growth of MAPbI_3_ Single Crystal

The MAPbI_3_ thin single crystals were grown by using the inverse temperature space-confined method in the hydrophobic substrates which were obtained by applying a hydrophobic reagent to the glass surface. Equal molars of PbI_2_ and MAI are dissolved in GBL and prepared as a 1.5 M MAPbI_3_ precursor solution and stirred for 2 hours at 70°C. The precursor solution was injected between the two hydrophobic substrates on the hot stage at 75°C, and then, the temperature was increased by 5°C every hour up to 120°C. The crystals were removed from the hot stage after growing to the suitable size.

### 4.3. Device Fabrication

To fabricate devices for SCLC measurement, 20 nm C60, 7.5 nm BCP, and 50 nm Cu were deposited sequentially on the surface of the SCs through a shadow mask with a 50 *μ*m channel width. To prepare devices for *E*_a_ measurement, 50 nm thick Au electrodes with a 50 *μ*m channel width were deposited on the surface of the SCs through a shadow mask. To fabricate X-ray detectors, 50 nm interdigital Au electrodes were deposited on the surface of the SCs through a shadow mask. For the MAI treatment, 1 mg mL^−1^ MAI solution in IPA was deposited on the SC surface by spin coating at 3000 r.p.m and then annealed at 50°C for 10 min on a hot plate. For the MAI-atomistic passivation, the excess MAI on the SC surface was washed away by spin coating IPA.

### 4.4. Material and Device Characterization

For the *E*_a_, SCLC, and *I* − *t* measurements of the SC devices, they were measured by a Keithley 2400 source meter in the N_2_-filled glove box. The XPS measurement were conducted with ESCALAB 250Xi X-ray photoelectron spectrometer. The PL spectra were measured by an optical fiber spectrometer QE65 Pro (Ocean Optics, U.S.A.) with 365 nm LED as the excitation source. The TRPL spectra were obtained by a HORIBA Scientific Fluoromax-4P instrument with a 370 nm pulse laser as the excitation source. The AFM measurement was carried out by a Bruker FastScan AFM system.

The X-ray detectors were measured in the open air. An Amptek Mini-X2 tube was used as the X-ray source. The X-ray source was collimated by a brass cylinder with a 2 mm diameter central hole in it, and the X-ray detector was placed very close to the outlet of the X-ray source. The dose rate of the X-ray source was tuned by changing the tube current, as well as by adding aluminum foils with various thicknesses as the filters. The actual X-ray dose rate on the device was carefully calibrated with a Radcal Accu-Gold+ 10X6-180 ion chamber dosimeter, by placing the dosimeter at the same position as that of the device to be measured. The photocurrent and dark current of the devices were recorded with a Keithley 2400 source meter. Based on the measured photocurrent *I*, the X-ray dose rate *D*, and the X-ray spot area *A*, the sensitivity *S* can be calculated based on the equation: *S* = *I*/*AD*. The noise of the devices was measured with an Agilent 35670A dynamic signal analyzer and a SRS 570 current preamplifier.

## Supplementary Materials

Supplementary Materials Figure S1: AFM characterization of MAPbI_3_ single crystal surface. The RMS values of the roughness of (a) untreated, (b) MAI treatment, and (c) atomistic passivation are 2.14 nm, 1.46 nm, and 1.42 nm, respectively. Figure S2: the single-log plots of the PL spectra. Figure S3: the attenuation coefficient of MAPbI_3_ as a function of incident photon energy. Figure S4: the X-ray response of the device on insulating glass substrates measured by successively turning on and off the X-ray source with the dose rate of 20.3 *μ*Gy_air_ s^−1^, which corresponds to a sensitivity of about 16 *μ*C Gy_air_^‐1^ cm^−2^ that comes from the contribution of the air ionization.
