# Supplementary figures and images for: Atomistic Surface Passivation of CH3NH3PbI3 Perovskite Single Crystals for Highly Sensitive Coplanar-Structure X-Ray Detectors
Source: Research (Wash D C). 2020 Sep 22;2020:5958243. doi: 10.34133/2020/5958243 (PMC7528034; doi:10.34133/2020/5958243)

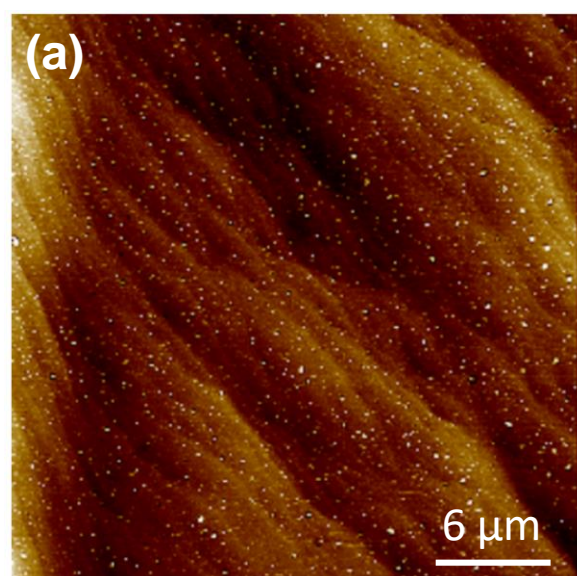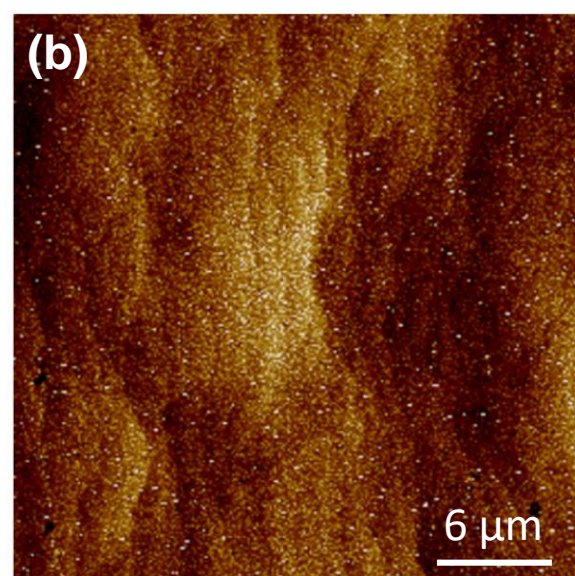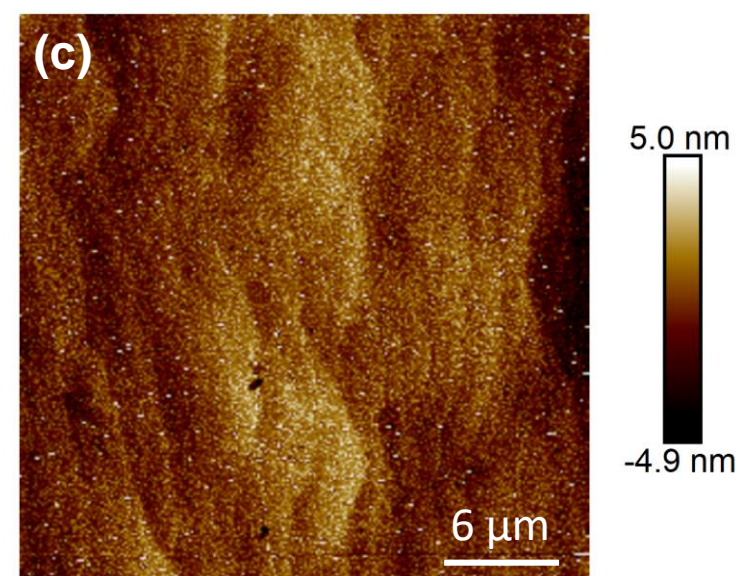

Supplement: Supplementary Materials — Figure S1: AFM characterization of MAPbI3 single crystal surface. The RMS values of the roughness of (a) untreated, (b) MAI treatment, and (c) atomistic passivation are 2.14 nm, 1.46 nm, and 1.42 nm, respectively. Figure S2: the single-log plots of the PL spectra. Figure S3: the attenuation coefficient of MAPbI3 as a function of incident photon energy. Figure S4: the X-ray response of the device on insulating glass substrates measured by successively turning on and off the X-ray source with the dose rate of 20.3 μGyair s−1, which corresponds to a sensitivity of about 16 μC Gyair‐1 cm−2 that comes from the contribution of the air ionization. [file 5958243.f1.zip › 5958243.f1/Figure S1.pdf]

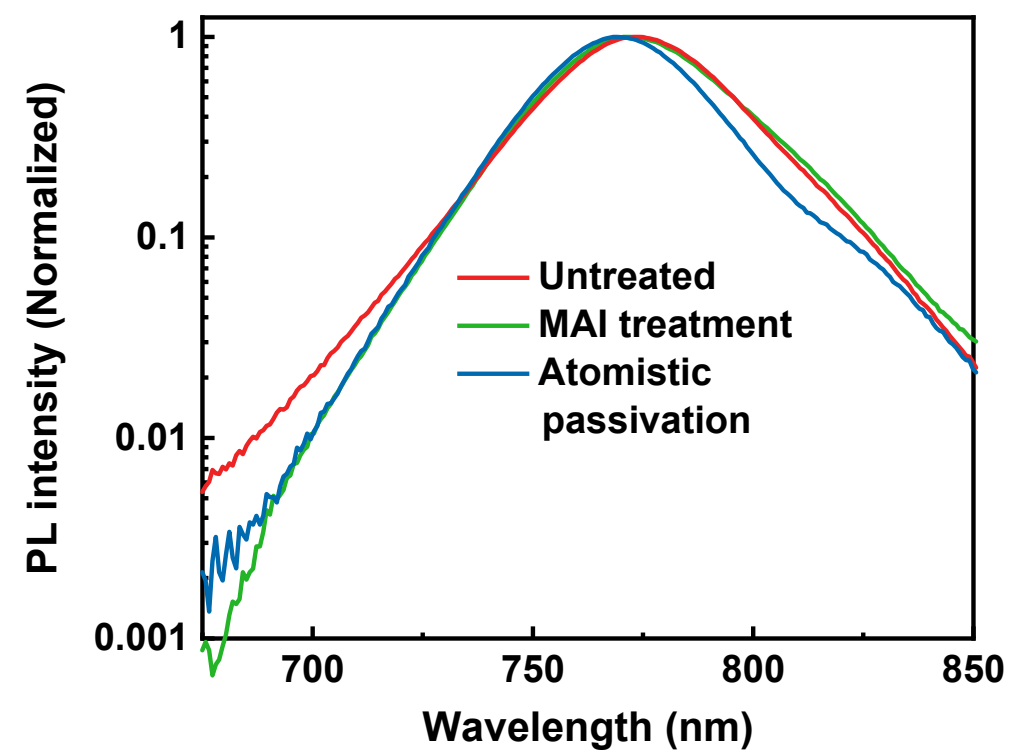

Supplement: Supplementary Materials — Figure S1: AFM characterization of MAPbI3 single crystal surface. The RMS values of the roughness of (a) untreated, (b) MAI treatment, and (c) atomistic passivation are 2.14 nm, 1.46 nm, and 1.42 nm, respectively. Figure S2: the single-log plots of the PL spectra. Figure S3: the attenuation coefficient of MAPbI3 as a function of incident photon energy. Figure S4: the X-ray response of the device on insulating glass substrates measured by successively turning on and off the X-ray source with the dose rate of 20.3 μGyair s−1, which corresponds to a sensitivity of about 16 μC Gyair‐1 cm−2 that comes from the contribution of the air ionization. [file 5958243.f1.zip › 5958243.f1/Figure S2.pdf]

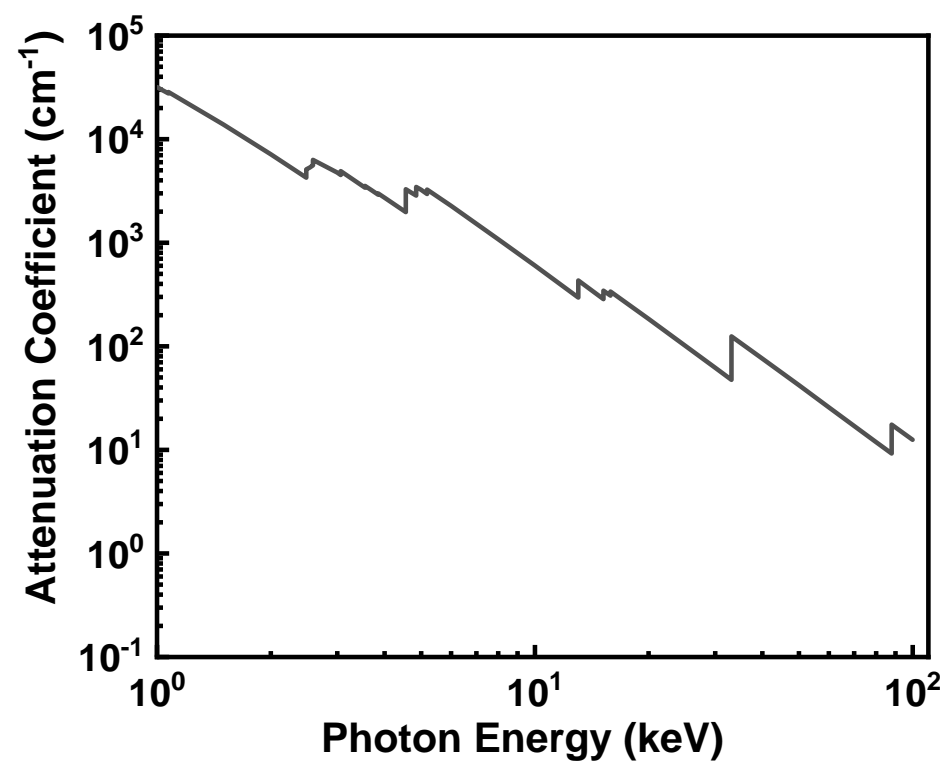

Supplement: Supplementary Materials — Figure S1: AFM characterization of MAPbI3 single crystal surface. The RMS values of the roughness of (a) untreated, (b) MAI treatment, and (c) atomistic passivation are 2.14 nm, 1.46 nm, and 1.42 nm, respectively. Figure S2: the single-log plots of the PL spectra. Figure S3: the attenuation coefficient of MAPbI3 as a function of incident photon energy. Figure S4: the X-ray response of the device on insulating glass substrates measured by successively turning on and off the X-ray source with the dose rate of 20.3 μGyair s−1, which corresponds to a sensitivity of about 16 μC Gyair‐1 cm−2 that comes from the contribution of the air ionization. [file 5958243.f1.zip › 5958243.f1/Figure S3.pdf]

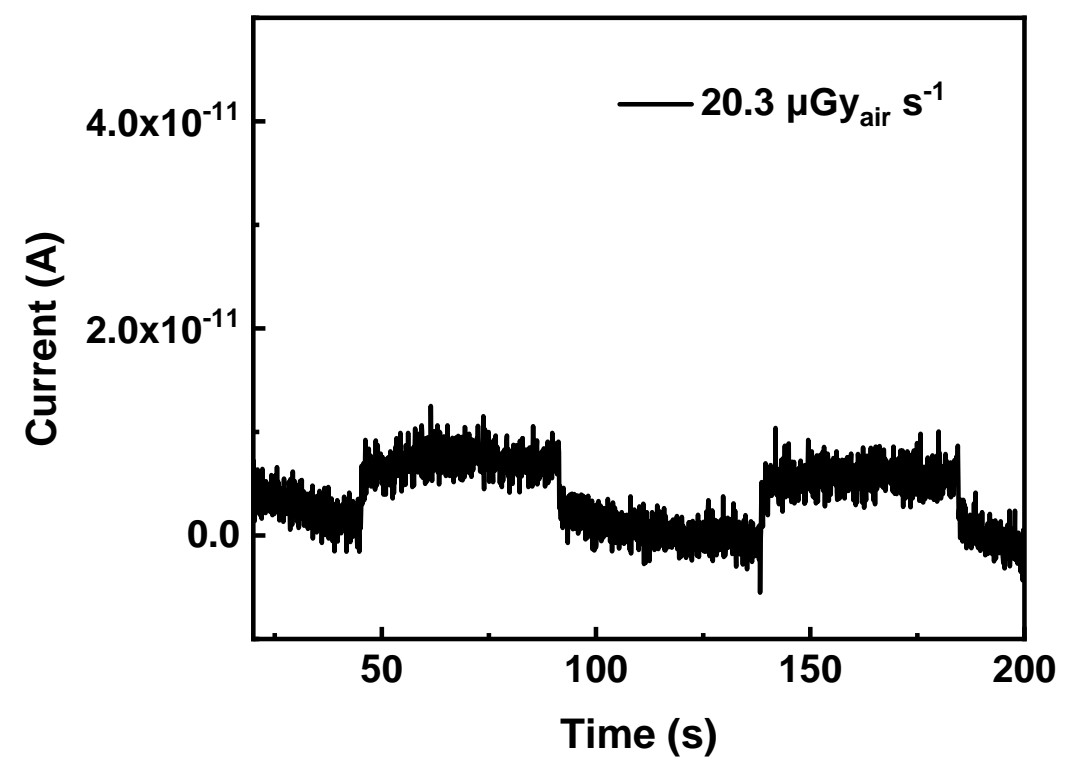

Supplement: Supplementary Materials — Figure S1: AFM characterization of MAPbI3 single crystal surface. The RMS values of the roughness of (a) untreated, (b) MAI treatment, and (c) atomistic passivation are 2.14 nm, 1.46 nm, and 1.42 nm, respectively. Figure S2: the single-log plots of the PL spectra. Figure S3: the attenuation coefficient of MAPbI3 as a function of incident photon energy. Figure S4: the X-ray response of the device on insulating glass substrates measured by successively turning on and off the X-ray source with the dose rate of 20.3 μGyair s−1, which corresponds to a sensitivity of about 16 μC Gyair‐1 cm−2 that comes from the contribution of the air ionization. [file 5958243.f1.zip › 5958243.f1/Figure S4.pdf]
